# Localized lichen planopilaris without patches: a multicenter retrospective study of 74-patients^؆^

**DOI:** 10.1016/j.abd.2026.501404

**Published:** 2026-06-18

**Authors:** Claudia Montoya, Maria Andrea Ocampo, Rita Fernanda Cortez de Almeida, André Luiz Vairo Donda, Michela Starace, Bianca Maria Piraccini, Gener Alejandro Mancilla, Lidia Rudnicka, Adriana Rakowska, Anna Waskiel-Burnat, Asmahane Souissi, Awatef Kelati, Kuzma Khobzei, Tatiana Siliuk, Andrei Doroshkevich, Renan Minotto, Cecilia Navarro Tuculet, Maria Eugenia Cappetta, Luis Enrique Sánchez-Dueñas, Maria Julia Mojardin-Lopez, Mariana Lavia, Boris Sanchez, Daniela Lynett, Sebastian Augusto Mercau, Aldo Gálvez-Canseco, David Saceda-Corralo, Carla Jorge Machado, Daniel Fernandes Melo

**Affiliations:** a Department of Dermatology, Universidad del Norte, Barranquilla, Colombia; b Private Dermatology Practice, Bogotá, Colombia; c Research Department, Advanced Institute of Trichology, Rio de Janeiro, RJ, Brazil; d Department of Experimental, Diagnostic and Specialty Medicine-Division of Dermatology, University of Bologna, Bologna, Italy; e Private Dermatology Practice, Bologna, Italy; f Group of Investigative Dermatology, Medicine Faculty, University of Antioquia, Medellín, Colombia; g Dermatology Section, Health Sciences School, Universidad Pontificia Bolivariana, Medellín, Colombia; h Department of Dermatology, Central Clinical Hospital, Ministry of the Interior, Warsaw, Poland; i Department of Dermatology, La Rabta Hospital, Tunis, Tunisia; j Faculty of Medicine of Tunis, University of Tunis El Manar, Tunis, Tunisia; k Dermatology Department, University Hospital Cheikh Khalifa, and the University Hospital Mohammed VI. Faculty of Medicine, Mohammed VI University of Health and Sciences, Casablanca, Morocco; l Private Practice, Kyiv, Ukraine; m Hair Treatment and Transplantation Center, Saint Petersburg, Russian Federation; n Center of Hair Treatment and Transplantation, St Petersburg, Russia; o Department of Dermatology, Hospital Santa Casa, Universidade Federal de Ciências da Saúde, Porto Alegre, RS, Brazil; p Dermatology Department, Hospital Italiano de Buenos Aires, Buenos Aires, Argentina; q Dermatologic Institute of Jalisco “Dr. José Barba Rubio”, University of Guadalajara, Jalisco, Mexico; r Universidad Autónoma de Sinaloa, Culiacán, Sinaloa, Mexico; s Private Dermatology Practice, Tricomed, Buenos Aires, Argentina; t Department of Dermatology, Universidad El Bosque, Bogotá, Colombia; u Private Dermatology Practice, Dermahair Center, Bucaramanga, Colombia; v Dermatology Department, Trichology Unit, Hospital Centenario, and Clínica de la Piel, Rosario, Argentina; w Private Dermatology Practice, Lima, Peru; x Department of Dermatology, University Hospital Ramón y Cajal, IRICYS, Madrid, Spain; y Preventive and Social Medicine Department, Universidade Federal de Minas Gerais, Belo Horizonte, MG, Brazil

Dear Editor,

Lichen planopilaris (LPP) is a primary lymphocytic cicatricial alopecia characterized by inflammation targeting the follicular infundibulum and isthmus, in which CD8+ T-cells attack autoantigens located in the follicular promontory, leading to irreversible follicular destruction.^1^, ^2^ Traditionally, it presents as well-defined alopecic patches with perifollicular erythema and scaling.^3^, ^4^ However, recent evidence has broadened the clinical spectrum of LPP, including diffuse non-patch variants that even mimic non-scarring alopecias.^5^ This article aims to describe epidemiological, clinical, trichoscopic, and histopathologic features of a localized non-patch form of LPP confined to specific scalp regions.

We performed a multicenter retrospective study involving 74 patients from 20 trichology referral centers. All cases were confirmed by trichoscopy and histopathology. Patients were excluded if they had androgenetic alopecia, frontal fibrosing alopecia, Fibrosing Alopecia in a Pattern Distribution (FAPD), or diffuse LPP. We compared our findings with the largest case series of diffuse variants of scalp LPP to date.^5^

Patients were mostly female (82.4%), with a mean age at onset of 41-years and a mean disease duration before diagnosis of 3.72-years. The vertex was most affected (82.4%). Scalp erythema was observed in 75.6% of cases. The predominant subjective symptom was mild to moderate pruritus (71.6%). The main trichoscopic features were perifollicular scaling (95.9%), perifollicular erythema (93.2%), and loss of follicular openings (73%). (Fig. 1A‒B). Histopathological examination revealed follicular dropout (100%), perifollicular concentric lamellar fibrosis (86.5%), reduction in the number of follicles (60.6%), and loss or reduction of sebaceous glands in 52.7% of patients. The most prescribed treatment was topical corticosteroids (98.6%), followed by topical tacrolimus (65.8%) and oral minoxidil (64.9%); Table 1, Table 2 provide the complete results.

**Fig. 1:**
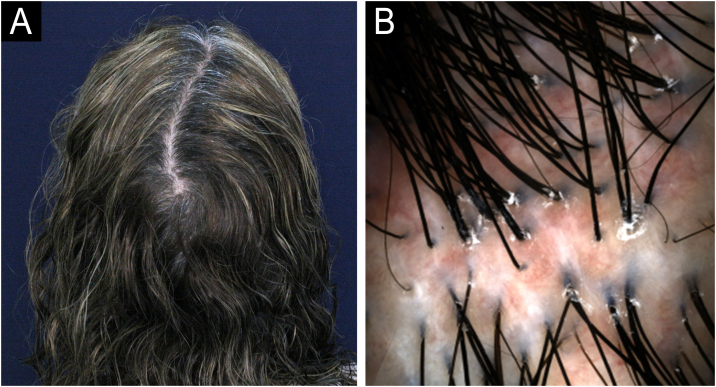
(A) Non alopecic patches in vertex area. (B) Perifollicular erythema and scaling and loss of follicular openings.

**Table 1:** Demographic and clinical characteristics of patients with lichen planopilaris.

Variables	Categories	Statistics	
Demographic Characteristics and Individual History			
- Female Gender: n (%) (n = 74)			
- Phototype: n (%) (n = 74)	*II*	16	(21.6)
	*III*	41	(55.4)
	*IV*	12	(16.2)
	*V*	5	(6.8)
- Body Mass Index: n (%) (n = 74)	Normal	67	(90.5)
	Overweight	7	(9.5)
- Family history of LPP: n (%) (n = 74)		7	(9.5)
- Menopause (n = 61): n (%) (n = 74)		26	(42.6)
- Age: mean (SD) // median // min; max (n = 74)		44.9 (13.8) // 44 // 14;74	
- Age at onset: mean (SD) // median // min; max (n = 71)		41.3 (13.8) // 42 // 13;71	
- Disease duration (years): mean (SD) // median // min; max (n = 71)		3.7 (3.9) // 3 // 0; 20	
			
Exposures and Habits			
- Sun exposure: n (%) (n = 74)		42	(56.8)
- Sun exposure minutes/day: mean (SD) // median // min; max (n = 29)		53.6(49.0) //30 // 15;240	
- Sunscreen use on scalp: n (%) (n = 74)		3	(4.1)
- Other photoprotection: n (%) (n = 74)		16	(21.6)
- Hair dye use: n (%) (n = 74)		37	(50.0)
- Chemical damage/discoloration: n (%) (n = 74)		12	(16.2)
- Smoking: n (%) (n = 74)		15	(20.3)
- Chemical hair straightening: n (%) (n = 74)		9	(12.2)
- Alcohol use: n (%) (n = 74)		7	(9.5)
- Hormone use: n (%) (n = 74)		7	(9.5)
			
Medical History and Comorbidities: n (%) (n = 74)			
- Autoimmune diseases		5	(6.8)
- Hashimoto's thyroiditis		3	(4.1)
- High blood pressure		3	(4.1)
- Hypercholesterolemia		2	(2.7)
- Other		15	(20.3)
Localization: n (%) (n = 74)			
- Vertex		36	(48.6)
- Vertex + mid scalp		25	(33.8)
- Other combinations		13	(17.6)
			
Clinical Symptoms and Signs: n (%)			
- Pruritus (n = 74)		53	(71.6)
*- Pruritus intensity (n = 53)*	*Mild*	29	(54.7)
	*Moderate*	20	(37.7)
	*Severe*	4	(7.5)
- Pain (n = 74)		28	(37.8)
*-- Pain intensity (n = 27)*	*Mild*	20	(74.1)
	*Moderate*	7	(25.9)
- Burning		23	(31.1)
*-- Burning intensity (n = 27)*	*Mild*	13	(61.9)
	*Moderate*	7	(33.3)
	*Severe*	1	(4.8)
- Positive pull test (n = 74)		12	(16.2)
- Disease spreading (n = 74)	*No*	52	(70.3)
	*Indeterminate*	10	(13.5)
	*Yes*	12	(16.2)
	
Disease activity score: mean (SD) // median// min; max (n = 71)		2.9 (1.8) // 2.6 // 0.3; 9.0

**Table 2:** Trichoscopic, histopathological and treatment characteristics of patients with lichen planopilaris.

**Variables**		**Statistics**	
**Trichoscopic Findings** (n = 74)		n	(%)
*Inflamatory Signs*			
-- Scalp erythema	*Absent*	14	(18.9)
	*Mild*	36	(48.6)
	*Moderate*	20	(27.0)
	*Severe*	4	(5.4)
-- Perifollicular erythema	*Absent*	5	(6.8)
	*Mild*	32	(43.8)
	*Moderate*	36	(49.3)
	*Severe*	1	(1.4)
-- Perifollicular scaling	*Absent*	3	(1.4)
	*Mild*	32	(43.2)
	*Moderate*	30	(40.5)
	*Severe*	9	(12.2)
-- Absent follicular openings		54	(73.0)
-- Perifollicular tubular casts		42	(56.8)
-- White structureless areas		36	(48.6)
-- Milky red areas		32	(43.2)
-- Fibrotic white dots		31	(41.9)
-- Arborizing vessels		25	(33.8)
-- Small tufts		15	(20.3)
-- Pili torti		14	(18.9)
-- Broken hairs		13	(17.6)
-- Scattered pigmentation		13	(17.6)
-- Loss of honeycomb pattern		11	(14.9)
-- Perifollicular gray to blue-gray structures		10	(13.5)
-- Hairpin vessels		9	(12.2)
-- Black dots		8	(10.8)
-- Red globules		7	(9.5)
-- Dotted vessels		6	(8.1)
-- Linear vessels		5	(6.8)
-- Perifollicular blue-gray dots		3	(1.4)
-- Target sign		3	(1.4)
-- Circular hairs		3	(1.4)
-- Interfollicular brown globules		3	(1.4)
**Histopathological Findings** (n = 74): n (%)			
-- Perifollicular lamellar fibrosis		64	(86.5)
-- Reduction in number of individual follicles		43	(58.1)
-- Loss/reduction of sebaceous glands		39	(52.7)
-- Basal layer vacuolar degeneration		30	(40.5)
-- Outer root sheath thining		24	(32.4)
-- Perifollicular mucin		8	(10.8)
-- Colloid bodies		7	(9.5)
**Treatments** (n = 74): n (%)			
-- Topical corticosteroids		73	(98.7)
-- Topical tacrolimus		48	(64.9)
-- Oral minoxidil		48	(64.9)
-- Tetracyclines		25	(33.8)
-- Hydroxychloroquine		20	(27.0)
-- Systemic steroids		15	(20.3)
-- Dutasteride		9	(12.2)
-- Methotrexate		3	(1.4)
-- Other (Pioglitazone/JAK inhibitors)		1	(1.4)

Starace et al. analyzed 40 patients with diffuse variants of scalp LPP.^5^ Our group was notably younger and predominantly premenopausal compared with both Lichen Planopilaris Diffuse Pattern (LPPDP) and Cicatricial Pattern Hair Loss (CPHL) – which might not represent a distinct disease entity but a post-inflammatory or residual form of cicatricial alopecia (Table 3). Our cohort showed milder symptoms (less pain/pruritus) and preserved sebaceous glands in 47.3% vs. 0% in both comparator groups, with moderate to severe hair loss (Table 3). Although the mean disease duration of 3.7-years supports an early or mild form in most patients, the presence of longer disease duration in isolated cases suggests that this localized, non-patch presentation may represent a stable, indolent phenotype within the LPP spectrum rather than a purely initial phase. These findings indicate less severe/chronic follicular injury. Unlike diffuse LPP, inflammation was localized ‒ predominantly at the vertex ‒ with lower activity (mean LPPAI 2.9), aligned with Miteva,^6^ who performed trichoscopy-guided biopsies in 43 patients presenting with scalp pruritus or sensitivity, but without obvious patches and presumed to have subtle or early-stage LPP. Notably, 70% showed no spread beyond the initial focus, and trichoscopy revealed early inflammatory signs before overt alopecia, consistent with a mild, early disease stage and supporting frequent use of topical-only therapy.

**Table 3:** Comparison of study findings (n = 74) with Starace et al. (2020) (n = 40) findings.

Variables	Present study finding	Comparison to Other Groups: Starace et al. (2020)	p-values for differences among groups
**Demographic characteristics**			
*Age (Mean)*	Youngest mean age (44.9 yrs)	Significantly younger than CPHL (56.9 yrs) and LPPDP (53.0 yrs)	<0.001
*Menopause*	Lowest rate of menopausal women (42.6%)	Significantly lower than CPHL (92.9%) and LPPDP (63.6%)	0.001
*Gender (Female %)*	Highest percentage of females (82.4%)	Higher than CPHL (70.0%) and LPPDP (55.0%)	0.034
**Clinical symptoms/Histopathology**			
*Pain*	Lowest prevalence (37.8%)	Significantly lower than LPPDP (100.0%) and CPHL (80.0%)	0.001
*Pruritus (Itching)*	Lowest prevalence (71.6%)	Significantly lower than LPPDP (100.0%) and CPHL (90.0%)	0.004
*Loss/Reduction of Sebaceous Glands*	Nearly half showed preservation (47.3%)	Both CPHL and LPPDP reported 0.0% preservation, with high rates of moderate/severe loss	0.001
**Trichoscopic findings**			
*Perifollicular Erythema*	Varied degree, with a high rate of moderate/severe (50.7%)	LPPDP was 100.0% mild (level 1); CPHL not reported	0.001
*Small Tufts*	Presence (20.3%)	Higher than CPHL (0.0%) but lower than LPPDP (45.0%)	0.001
*Broken Hairs*	Presence (17.6%)	Higher than CPHL (0.0%) but lower than LPPDP (45.0%)	0.001

Notes: CPHL, Cicatricial Pattern Hair Loss; LPPDP, Lichen Planopilaris Diffuse Pattern.

Sun exposure was frequent (56.8%) with a mean duration of 53.6 minutes per day. However, scalp photoprotection was minimal (4.1%), and only 21.6% reported any form of physical protection. The vertex, a chronically sun-exposed area, could be predisposed to UV-induced disruption of follicular immune privilege, promoting localized autoimmune inflammation in susceptible individuals.^7^, ^8^ The use of hair dye and chemical hair straightening reinforces the hypothesis that chronic chemical and photo-oxidative stress may act as triggering factors.^9^, ^10^

FAPD is an important differential diagnosis, though non-patch LPP does not present miniaturized hair. Other inflammatory scalp conditions should also be ruled out as they depict different erythema and scaling patterns.

To our knowledge, this is the first case series focused exclusively on the localized non-patch form of LPP. Moreover, our study pointed out that this condition may represent an early stage of classic or diffuse LPP. Finally, recognizing this presentation is crucial, as timely anti-inflammatory treatment may halt disease progression and scarring. Trichoscopy-guided biopsy of symptomatic yet non-alopecic areas can confirm diagnosis and guide intervention. Further studies are warranted to determine whether this form remains localized or progresses to classic patchy or diffuse non-patch LPP, and to clarify the roles of UV exposure and cosmetic habits in disease onset.

## ORCID ID

Maria Andrea Ocampo: 0000-0002-8857-6180

Rita Fernanda Cortez de Almeida: 0000-0001-7904-998X

Michela Starace: 0000-0002-3981-1527

Bianca Maria Piraccini: 0000-0001-6537-9689

Gener Alejandro Mancilla: 0000-0002-3333-503X

Lidia Rudnicka: 0000-0002-8308-1023

Adriana Rakowska: 0000-0001-6117-3704

Anna Waskiel-Burnat: 0000-0003-4984-3589

Asmahane Souissi: 0000-0002-0058-0645

Awatef Kelati: 0000-0003-2570-4584

Kuzma Khobzei: 0009-0002-2879-9240

Tatiana Siliuk: 0000-0001-9118-2396

Andrei Doroshkevich: 0009-0009-8093-7269

Renan Minotto: 0000-0002-1451-0461

Cecilia Navarro Tuculet: 0000-0003-0613-9962

Maria Eugenia Cappetta: 0009-0006-8798-789X

Luis Enrique Sánchez-Dueñas: 0000-0002-2136-1230

Maria Julia Mojardin-Lopez: 0009-0004-4252-7537

Mariana Lavia: 0009-0008-8134-6113

Boris Sanchez: 0000-0002-6096-136X

Daniela Lynett: 0000-0003-1338-4384

Sebastian Augusto Mercau: 0009-0001-2384-5159

Aldo Gálvez-Canseco: 0000-0002-8644-634X

David Saceda-Corralo: 0000-0001-6315-5462

Carla Jorge Machado: 0000-0002-6871-0709

Daniel Fernandes Melo: 0000-0002-8807-2556

## Financial support

None declared.

## Authors’ contributions

Claudia Montoya, Maria Andrea Ocampo, Rita Fernanda Cortez de Almeida, André Luiz Vairo Donda: Data collection, analysis and interpretation of data; writing of the manuscript and critical review of important intellectual content; data collection, analysis and interpretation, critical review of the literature; final approval of the final version of the manuscript.

Michela Starace, Bianca Maria Piraccini, Gener Alejandro Mancilla, Lidia Rudnicka, Adriana Rakowska, Anna Waskiel-Burnat, Asmahane Souissi, Awatef Kelati, Kuzma Khobzei, Tatiana Siliuk, Andrei Doroshkevich, Renan Minotto, Cecilia Navarro Tuculet, Maria Eugenia Cappetta, Luis Enrique Sánchez-Dueñas, Maria Julia Mojardin-Lopez, Mariana Lavia, Boris Sanchez, Daniela Lynett, Sebastian Augusto Mercau, Aldo Gálvez-Canseco, David Saceda-Corralo: Data collection; intellectual participation in the propaedeutic and therapeutic conduct of the studied cases; final approval of the final version of the manuscript.

Carla Jorge Machado: Statistical analysis; critical review of important intellectual content; final approval of the final version of the manuscript.

Daniel Fernandes Melo: The study concept and design; data collection, analysis and interpretation of data; critical review of important intellectual content; intellectual participation in the propaedeutic and therapeutic conduct of the studied cases; critical review of the literature; final approval of the final version of the manuscript.

## Research data availability

The entire dataset supporting the results of this study was published in this article.

## Conflicts of interest

None declared.
